# Case Report: Pituitary metastasis from prostate adenocarcinoma: an integrated diagnosis via ^68^Ga-PSMA PET/CT and methylome analysis

**DOI:** 10.3389/fmed.2025.1618256

**Published:** 2025-06-18

**Authors:** N. Bianchetto Wolf, I. C. Mainta, A. Fitsiori, G. Colarusso, P. Tsantoulis, K. L. Egervari, V. Garibotto

**Affiliations:** ^1^Division of Nuclear Medicine and Molecular Imaging, Geneva University Hospitals, Geneva, Switzerland; ^2^Division of Neuroradiology, Geneva University Hospitals, Geneva, Switzerland; ^3^Division of Oncology, Geneva University Hospitals, Geneva, Switzerland; ^4^Division of Clinical Pathology, Geneva University Hospitals, Geneva, Switzerland

**Keywords:** prostate cancer, pituitary metastasis, PSMA PET/CT, MRI, DNA methylation profiling

## Abstract

**Background:**

Pituitary metastases are rare, accounting for only 1% of all pituitary tumor resections. Prostate adenocarcinoma, a common malignancy in men, seldom metastasizes to the pituitary gland, with only a few reported cases. Given their rarity and non-specific clinical presentation, pituitary metastases are often mistaken for primary sellar lesions. Advanced imaging techniques, including Prostate-Specific Membrane Antigen (PSMA) positron emission tomography (PET)/Computed Tomography (CT) and molecular diagnostics, such as DNA methylation profiling, can aid in accurate diagnosis and differentiation from pituitary adenomas.

**Case presentation:**

We report the case of a 71-year-old male with a history of prostate adenocarcinoma who presented with biochemical recurrence and underwent PSMA PET/CT imaging, revealing intense tracer uptake in the pituitary gland. Magnetic Resonance Imaging (MRI) findings were suggestive of a pituitary macroadenoma, and the patient developed bitemporal hemianopia, necessitating transsphenoidal surgical resection. Histopathological and immunohistochemical analyses were not compatible with a primary pituitary lesion, prompting further investigation via DNA methylation profiling. The analysis revealed a DNA-methylation signature consistent with prostate carcinoma, confirming pituitary metastasis. The patient subsequently received systemic treatment with androgen deprivation therapy, abiraterone, and docetaxel, achieving an excellent biochemical and imaging response.

**Conclusion:**

This case highlights the importance of considering metastatic prostate cancer in the differential diagnosis of pituitary lesions, particularly when PSMA PET/CT shows focal uptake in atypical locations. Integration of histopathological, immunohistochemical, and molecular techniques, such as DNA-methylation profiling, was essential for confirming the diagnosis. Clinicians should remain vigilant for atypical metastatic presentations and leverage advanced diagnostic tools to ensure accurate diagnosis and optimal patient management.

## Introduction

Pituitary metastases are a rare occurrence, accounting for only 1% of all pituitary tumor surgical resections (1). Autopsy studies suggest that the pituitary gland is involved in metastasis in an even smaller percentage of cases, with an estimated incidence of approximately 0.6% (2).

Despite their rarity, these metastases are clinically significant due to the gland’s critical role in endocrine function. Involvement of the pituitary can lead to a wide range of symptoms (3).

Patients with pituitary metastases may present with signs of pituitary dysfunction, such as hypopituitarism, which manifests as symptoms such as fatigue, weight loss, and sexual dysfunction. Alternatively, symptoms may result from mass effects, including headaches and visual disturbances. A classic finding is visual field defects, particularly bitemporal hemianopia, due to compression of the optic chiasm by the expanding metastatic lesion. Central diabetes insipidus (DI) is also relatively common in pituitary metastases. Given the rarity and varied clinical presentation of these lesions, they are often mistaken for more common conditions, such as primary pituitary adenomas or other sellar masses, making timely diagnosis challenging.

Prostate adenocarcinoma is one of the most frequently diagnosed cancers in men, with an estimated 1.4 million new cases worldwide annually (4). This cancer typically metastasizes to bones (particularly the spine, pelvis, and femur) and lymph nodes and more rarely to distant organs such as the liver or lungs. Metastasis of prostate cancer to the pituitary gland is exceedingly rare, with only a limited number of cases reported in the literature: eight cases documented from 1957 to 2018, none of which included PET PSMA imaging (5).

Given the rarity of pituitary metastases, it is crucial to distinguish them from more common pituitary lesions, such as adenomas, craniopharyngiomas, or Rathke’s cleft cysts. Imaging techniques such as CT and MRI are essential for detecting pituitary lesions. MRI is the preferred modality for investigating pituitary pathologies due to its high spatial resolution and excellent soft tissue contrast (6). In recent years, positron emission tomography (PET) imaging using prostate-specific membrane antigen (PSMA) radiotracers has emerged as a valuable diagnostic tool in detecting prostate cancer metastases, including those in rare locations such as the pituitary gland.

Histopathological analysis is critical for confirming the diagnosis of pituitary metastasis. This typically involves biopsy and examination of tissue to assess the tumor’s nature and its potential origin. Morphological and immunohistochemical studies are essential and usually sufficient to differentiating metastases from primary pituitary adenomas, with markers such as PSMA, P501s, PHAP, and prostate-specific antigen (PSA) helping to confirm a prostatic origin of the metastatic lesion. In some cases, however, molecular analyses may also be necessary to definitively confirm the origin of a secondary lesion.

## Case description

A 71-year-old male patient had a history of Gleason 4 + 3 prostate adenocarcinoma, initially diagnosed through prostate biopsy, which revealed a carcinoma with perineural invasion. Imaging studies, including MRI of the prostate and ^18^F-choline PET/CT, indicated extensive tumor infiltration of the left prostate lobe, extending to the apex and base of the prostate, with strong suspicion of extracapsular extension and infiltration of the left seminal vesicle. Regional lymph node metastasis was strongly suspected, although no bone lesions were observed at that time (TNM classification at diagnostic: T3b N1 M0). The patient underwent locoregional radiotherapy in 2019 with a Simultaneous Integrated Boost, delivering 44 Gy to elective pelvic lymph nodes, 56 Gy to pelvic lymphadenopathy, and 60 Gy to the prostate and seminal vesicles, associated with leuprorelin treatment from 2019 to 2020.

**Figure 1:**
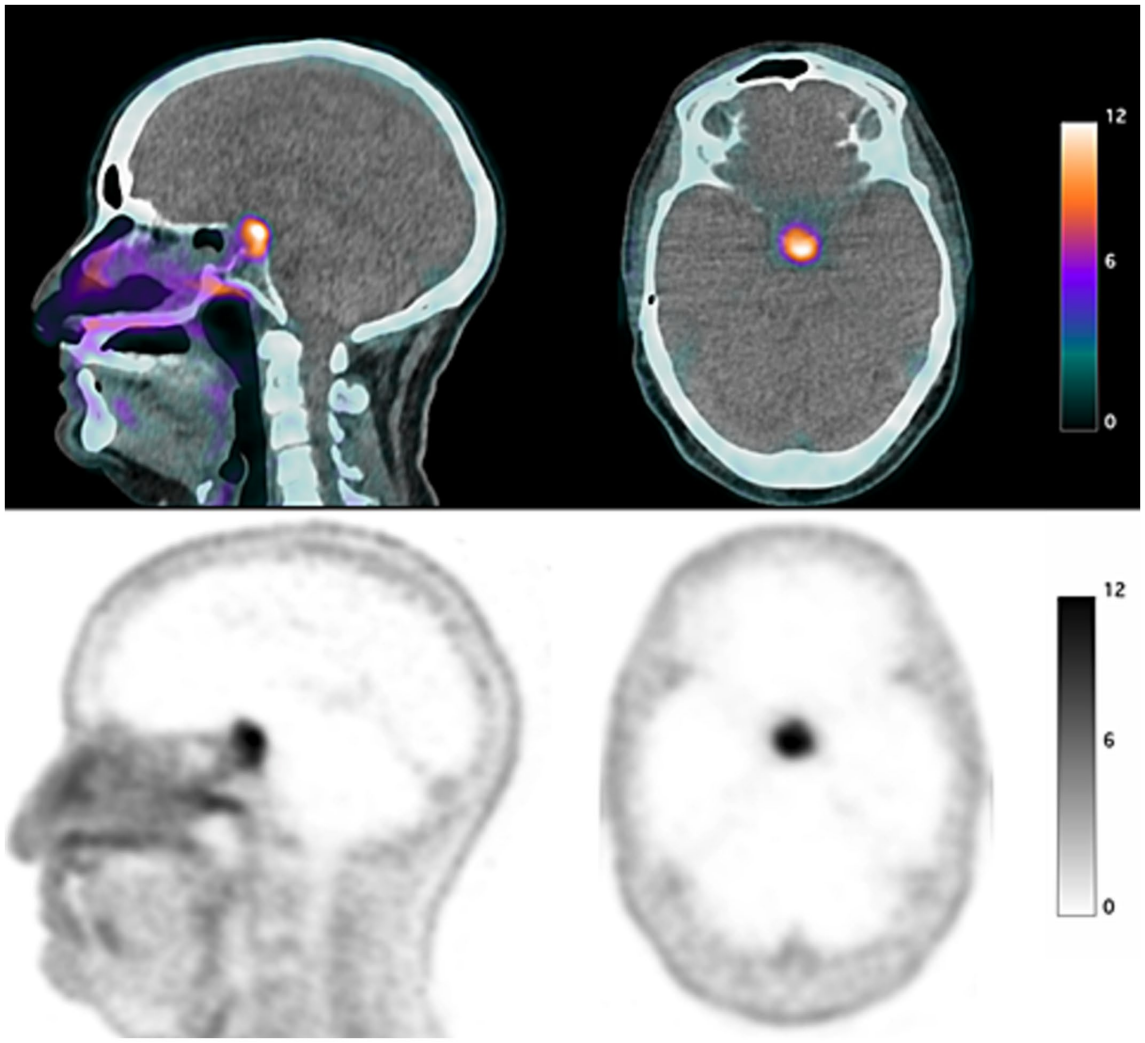
PET/CT showing an intense unhabitual fixation centered on the sellar region. Upper part: Fusion PET/CT axial and coronal; Lower part: PET axial and coronal.

In July 2021, the patient presented with biochemical recurrence as indicated by an elevated PSA, prompting further investigation. A PET ^68^Ga-PSMA-11 scan was conducted, revealing a focally increased uptake of the pituitary gland (SUV_max_: 17.1) with no other locoregional or distant focal uptake (Figure 1). This finding was considered atypical for prostate cancer metastasis, as pituitary involvement is rare in this context.

**Figure 2:**
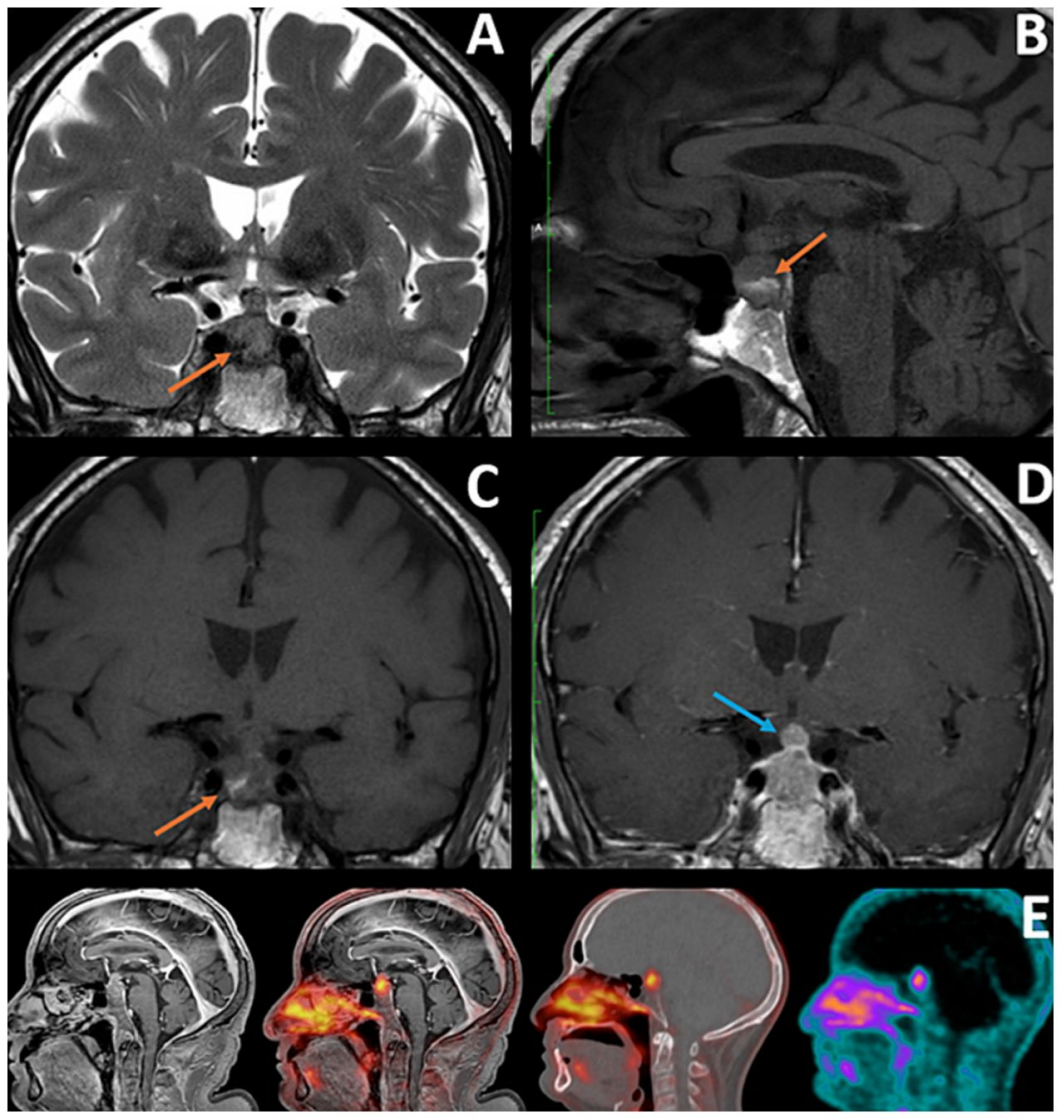
**(A–C)** Coronal T2, Sagittal, and Coronal T1: Orange arrows showing hemorrhagic component within the lesion, with hypointense signal in T2 WI and hyperintense signal in T1 WI (consistent with subacute character), which could be consistent with recent apoplexy of pre-existing pituitary adenoma (factor contributing to the initial misdiagnosis). **(D)** Coronal T1 with Gd: Blue arrow showing a nodular component of the lesion at the level of the pituitary stalk, a feature that, according to some researchers, may facilitate correct diagnosis of pituitary metastases. **(E)** IRM T1 TFE Gd; Fusion image PET/MRI; fusion image PET/CT; PET.

Following these findings, the patient underwent pituitary MRI (Figure 2), which showed an intra-sellar tissue lesion extending to the supra-sellar region, with a mass effect to the optic chiasma and in close proximity to internal carotid arteries, displaying heterogeneous signal intensity on T1- and T2-weighted MRI, with hemorrhagic components and mildly heterogeneous contrast enhancement. These imaging findings were compatible with a pituitary macroadenoma.

Due to the onset of bitemporal hemianopia detected during an ophthalmologic examination, a transsphenoidal surgical resection was decided. The procedure was performed in December 2021 without complications.

Despite the clinical suspicion of a pituitary adenoma, histological examination and immunohistochemical profiling failed to confirm a typical pituitary origin. In particular, the morphology revealed a glandulo-papillary proliferation, with tumor cells showing apical cytoplasmic snouts and vesicular nuclei with a nucleole. The immunohistochemical stains were positive for pan-keratine and synaptophysin stains, weakly positive for CK20, and negative for CK7, as well as for all hypophyseal hormones and SF-1. Prostate-specific stains, including PSA, PHAP, and P501S, showed only focal apical positivity in some cells, of which the specificity was questionable. The histopathological findings from the pituitary biopsy were, therefore, suggestive of a pituitary tumor with neuroendocrine differentiation, and the lack of expression of pituitary hormones raised the possibility of a secondary lesion.

DNA methylation profiling was performed using the Infinium MethylationEPIC Array. The data analysis was carried out on the EpiDiP server at the University Hospital of Basel (7), enabling the comparison of the methylation profile of our case against a reference cohort of over 15,000 annotated, publicly available human tumors.

**Figure 3:**
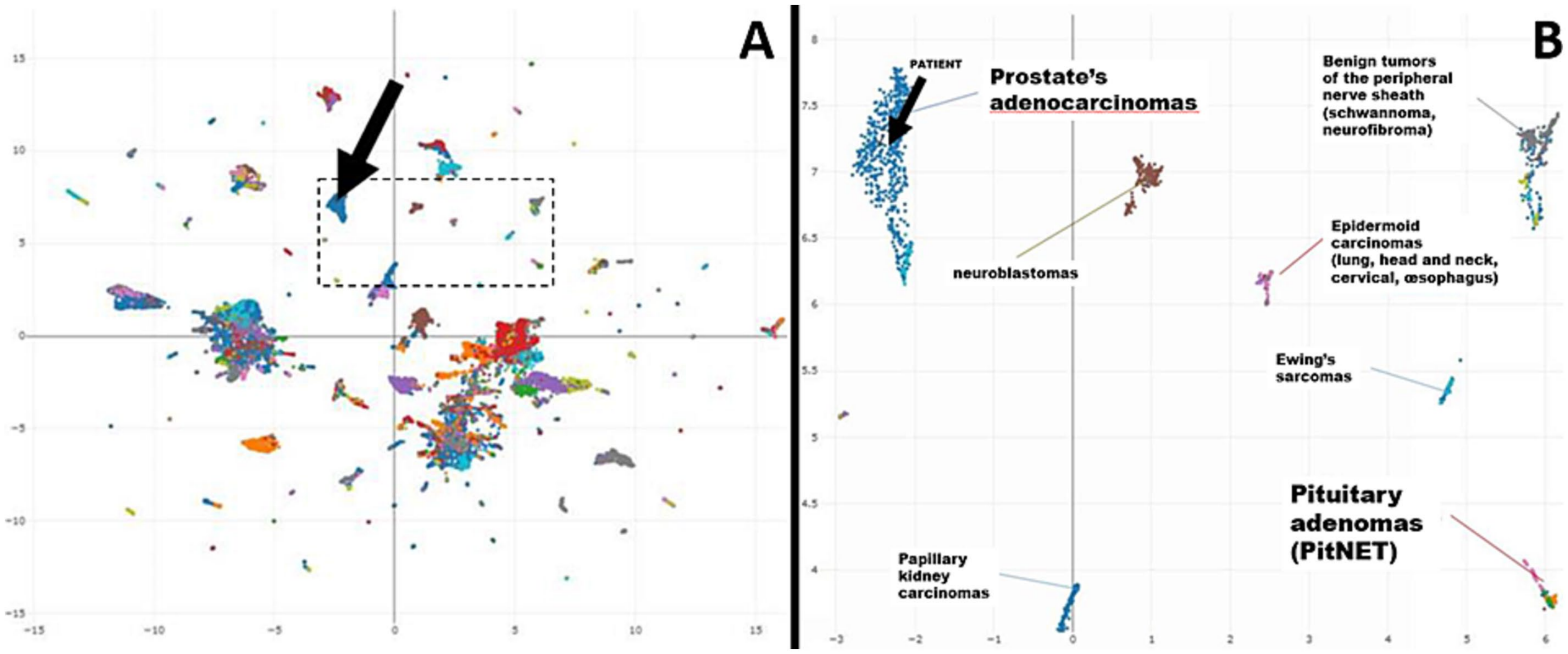
**(A)** Uniform manifold approximation and projection (UMAP) plot—Overview (25 k probes) (black arrow: patient’s case). **(B)** Uniform manifold approximation and projection (UMAP) plot—Excerpt (black arrow: patient’s case).

**Figure 4:**
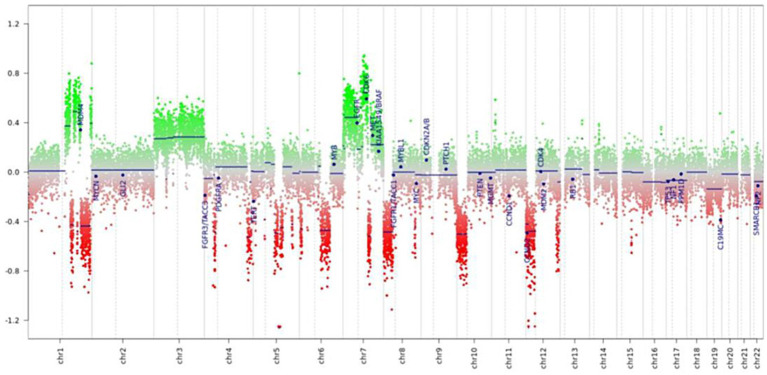
Prediction of genomic profile (copy number variations).

This analysis revealed that the methylation profile most closely resembled that of prostate carcinomas (Figure 3). In addition, the analysis showed chromosomal alterations commonly associated with prostate cancer, such as losses on chromosomes 8p, 10p, and 12p, and focal losses on chromosomes 1q, 4p, and 5q21.3 (CHD1) (Figure 4). The loss of CHD1 is significant as it has been linked to early resistance to anti-androgen treatment in prostate cancer.

The results of the histopathological examination, immunohistochemical profiling, and methylation analysis led to the final diagnosis of pituitary metastasis from prostate adenocarcinoma. The methylation profile provided the crucial confirmation of metastatic disease origin.

The patient subsequently underwent systemic treatment with hormone deprivation therapy and abiraterone since March 2022, along with 6 cycles of docetaxel chemotherapy from May to August 2022, achieving an excellent biochemical (indetectable PSA until June 2024) and imaging response.

This case underscores that, despite their rarity, prostate metastases to the pituitary should be considered in the differential diagnosis of focally increased uptake. While pituitary metastases are rare, this case demonstrates the value of comprehensive diagnostic techniques, including molecular analyses, to accurately characterize metastatic disease and guide appropriate treatment.

## Discussion

The patient presented with a metastatic recurrence of prostate adenocarcinoma, showing a significant PSMA uptake (Standardized Uptake Value Maximum, SUV_max_: 17.1) in an atypical location, the pituitary gland. This uptake was notable, with SUV_max_ value indicative of an abnormal focal retention, raising concerns about the metastatic spread in this region.

MRI characteristics could lead to diagnostic challenges. According to Yuzkan et al., a rapid growth, nodular expansion of the pituitary stalk, and a history of cancer could be useful for differentiating between pituitary metastases and adenomas (8).

Although the uptake of PSMA in the pituitary gland is uncommon, it has been reported in some studies involving pituitary adenomas. A recent case documented in the literature involved a 76-year-old man with metastatic prostate cancer, where the PSMA-PET/CT scan revealed mild uptake in the pituitary (SUV_max_: 1.9) despite a significant ^18^F-FDG accumulation (SUV_max_: 13.9) (9). Furthermore, a study by McLaughlin et al. analyzed 2,763 patients who underwent PSMA PET/CT scans, identifying 33 incidental PSMA-avid brain and head lesions, including pituitary macroadenomas (6%). Additionally, one non-PSMA-avid pituitary macroadenoma was detected. This study highlighted that pituitary adenomas exhibit low PSMA uptake, with a mean SUV_max_ of 4.45 (10). These findings highlight that while PSMA uptake in pituitary adenomas can occur, it is generally of lower intensity than prostate cancer secondary localization.

In this case, the histopathological findings from the pituitary biopsy, lacking the expression of pituitary hormones, raised the possibility of a metastatic tumor with neuroendocrine differentiation. To confirm this diagnosis and rule out the possibility of a pituitary adenoma without hormone expression (“null cell adenoma”), we decided to perform DNA methylation profiling. As DNA methylation is a stable epigenetic fingerprint of cellular origin, this technique can help distinguish between different types of tumors, even in cases where traditional histopathology and immunohistochemistry do not provide conclusive results. DNA methylation profiling has not only become the gold standard for the analysis of tumors of the central nervous system (11) but has also been developed for the classification of sarcomas (12–14) and metastatic tumors of unknown primary (7, 15). The analysis of raw methylation data from the EPIC Array (Illumina) on the publicly available EpiDiP server (16) is a Swiss-developed, user-friendly alternative to well-established classifiers (11, 12), which may help to decipher the origin of metastatic tumors in selected cases and after internal validation could be used in routine clinical practice (7). EpiDiP allows an unbiased comparison of a given sample to a large set of annotated tumor samples with known origins. The similarity of a given case to the reference data can be visually judged after integration in a UMAP, which, in our case, demonstrated a very high epigenetic similarity to carcinomas of prostatic origins. These findings encouraged the reconsideration of the focal apical positivity of prostate-specific immunostains. Our case demonstrates that DNA methylation provides an additional layer of information—allowing a more accurate interpretation of the tumor’s origin, complementing morphological and immunohistochemical analyses.

In addition to this, the methylation analysis allows the prediction of copy number variations (CNVs) and, in our case, revealed several significant chromosomal gains and losses, further supporting the diagnosis of metastasis from prostate cancer. These included a gain of chromosomes 3 and 7, with CDK6 (cyclin-dependent kinase 6) being involved, and a loss of the arms of chromosomes 8p, 10p, and 12p, all of which are frequently seen in prostate cancer. Moreover, multiple focal heterozygous losses were detected on chromosomes 1q, 4p, 4q, 6p, 6q, 7q, 8q, 12q, and 15q, and a homozygous focal loss on chromosome 5q21.3 involving the CHD1 gene. Homozygous deletion of CHD1 is relatively rare, found in approximately 11% of Caucasian patients, and has been associated with rapid disease progression (17). Interestingly, our patient has benefited from a long duration of response to triplet therapy with androgen deprivation, abiraterone, and docetaxel. Prospective clinical trials are required to confirm the influence of CHD1 deletion on the response to castration, abiraterone, enzalutamide, and other anti-androgen therapies in both hormone-sensitive and resistant prostate cancer (18).

In summary, PSMA PET/CT imaging may play a role in differentiating pituitary metastases from adenomas. In cases where PET/CT reveals a specific and intense radiotracer uptake, a metastatic lesion should be strongly suspected, as the limited literature available on this topic reports low uptake in pituitary adenomas (9, 10). The integration of molecular techniques, such as DNA methylation profiling, with traditional diagnostic methods can ultimately aid in confirming atypical metastases, such as pituitary involvement from prostate adenocarcinoma. The combination of multiple diagnostic approaches enhances accuracy and supports better-informed treatment decisions, ultimately improving patient management.

## Conclusion

This is the first reported case of pituitary metastasis from prostate adenocarcinoma diagnosed using PSMA PET/CT imaging in the literature. It emphasizes the importance of considering metastatic prostate cancer in the differential diagnosis of pituitary lesions, especially when PET-CT shows focal hyperfixation in unusual locations.

The integration of advanced diagnostic techniques, including histopathological evaluation and molecular analysis, such as DNA-methylation profiling, was essential in confirming the diagnosis of pituitary metastasis from prostate adenocarcinoma. This approach not only improved diagnostic accuracy but also provided insights into the genetic alterations associated with prostate cancer metastasis. Clinicians should be aware of the potential for metastasis in atypical sites, and the use of comprehensive diagnostic tools is crucial for appropriate diagnosis and management, particularly when managing complex cases of metastatic prostate cancer.

## Data Availability

The original contributions presented in the study are included in the article/supplementary material, further inquiries can be directed to the corresponding author.
